# Value of late gadolinium enhancement by magnetic resonance in patients with cardiac sarcoidosis: characteristic findings and clinical utility

**DOI:** 10.1186/1532-429X-11-S1-P260

**Published:** 2009-01-28

**Authors:** Eri Watanabe, Fumiko Kimura, Michiaki Hiroe, Takatomo Nakajima, Ayako Nakamura, Noriko Nishii, Masatoshi Kawana, Nobuhisa Hagiwara

**Affiliations:** 1 Institute of Geriatrics, Tokyo Women's Medical University, Shibuya-ku, Tokyo, Japan; 2 Saitama Medical University International Medical Center, Saitama, Japan; 3 International Medical Center of Japan, Tokyo, Japan; 4 Tokyo Women's Medical University, Tokyo, Japan; 5 Tokyo Women's Medical University, Aoyama Hospital, Tokyo, Japan

**Keywords:** Cardiac Magnetic Resonance, Late Gadolinium Enhancement, Cardiac Sarcoidosis, Septal Wall, Late Gadolinium Enhancement Image

## Introduction

Although cardiac involvement is an important prognostic factor in patients with sarcoidosis, cardiac sarcoidosis (CS) is often difficult to diagnose. Cardiac magnetic resonance (CMR) imaging with late gadolinium enhancement (LGE) is considered to be useful in identifying CS early.

## Purpose

We investigated LGE characteristics in patients with CS.

## Methods

The study included 17 patients who were diagnosed with CS by Japanese Ministry of Health and Welfare criteria and underwent CMR. Among the 17, 15 had LGE and were evaluated retrospectively. We obtained LGE images (short-axis, vertical long-axis, and 4-chamber views) using an inversion recovery segmented gradient-echo sequence 10 minutes after we administered gadolinium-DTPA (0.15 mmol/Kg or 20 mL). We analyzed patterns (patchy or band-like appearance) and locations of LGE in LV using a 17-segment model. We also assessed the extent of LGE in each segment of LV (subepicardial, midwall, subendocardial and transmural). We evaluated relationships between LGE characteristics and LV function using cine MR images.

## Results

We observed band-like LGE with distinct margin in 14 patients and patchy LGE in one. The band-like pattern correlated well with pathological findings in biopsy-proven CS. Transmural LGE (T-LGE) was observed in 7 patients. All patients with T-LGE also had subepicardial lesion. In the patients without T-LGE, LGE was most common in the subepicardial layer (74% of enhanced segments) and most frequently observed in the basal septal wall, especially on the RV side. The number of LGE segments of the patients with T-LGE was significantly larger than that of patients without T-LGE (14.0 ± 2.0 versus 4.3 ± 3.3 segments, p < 0.0001). The ejection fraction of the LV (LVEF) of the patients with T-LGE was significantly lower than that of patients without T-LGE (19.0 ± 5.9% versus 50.8 ± 6.5%, p < 0.0001), and the end-diastolic volume of the LV of the patients with T-LGE was significantly larger than that of patients without T-LGE (236.4 ± 50.6 versus 96.5 ± 9.7 mL, P < 0.0001).

## Conclusion

Characteristic LGE pattern and location allow accurate diagnosis of CS. CMR with LGE is useful for diagnosing CS and predicting LV function.

